# Race, Crime, and Lending Risk in Chicago: The Relevance of Crime and Disorder for HOLC’s Neighborhood Assessments

**DOI:** 10.1007/s12552-025-09442-4

**Published:** 2025-08-27

**Authors:** Megan Evans

**Affiliations:** 1 Max Planck Institute for Demographic Research, Rostock, Germany; 2 Max Planck - University of Helsinki Center for Social Inequalities in Population Health, Rostock, Germany

**Keywords:** Institutional racism, Lending risk, Perceived crime and disorder

## Abstract

While scholars have documented the importance of race for decisions on lending risk and value in the U.S. housing market, less is known about how crime shaped lending risk assessments or how a neighborhood’s racial composition influenced appraisers’ perceptions of crime and disorder. Drawing on the Home Owners’ Loan Corporation (HOLC) residential security maps, this study examines appraisers’ narratives around neighborhood crime and disorder, how observed neighborhood conditions shaped these narratives, and how both observed crime and perceptions of disorder influenced decisions on lending risk. Using the case of Chicago, this study integrates multiple historical datasets, including the HOLC residential security maps and their corresponding neighborhood area descriptions, the 1940 Census, and data on criminal activity reflected through Clifford Shaw et al.’s residence of male offenders map and Frederic Thrasher’s gangland activity map. Findings suggest that perceptions of crime and disorder are largely driven by a neighborhood’s Black racial composition, independent of observed measures of crime. While both observed crime and a neighborhood’s Black racial composition predicted lending risk assessments, appraisers’ perceptions of disorder did not. The results indicate that although HOLC appraisers’ perceptions of crime and disorder were racially motivated, their biased perceptions did not exert a unique, independent influence on their decisions to redline Black neighborhoods. Rather, racial discrimination was already explicitly embedded into their neighborhood valuation practices. This study provides new insights into the historical roots of neighborhood stigmatization and institutional disinvestment.

**Supplementary Information:**

The online version contains supplementary material available at 10.1007/s12552-025-09442-4.

## Introduction

Perceptions of crime and disorder are important determinants of how individuals interact with the urban environment, shaping where individuals decide to conduct their routine activities, start businesses, and buy homes (Doran & Burgess, 2012; Krysan & Crowder, 2017; Lewis et al., 2011; Sampson, 2012). Disorder serves as a signal of neighborhood status and desirability (Booth, 1889; Goffman, 1963; Hunter, 1985; Jacobs, 1961; Logan & Collver, 1983; Semyonov & Kraus, 1982), yet often when individuals perceive a neighborhood as disorderly it is more reflective of racial prejudices than actual observed conditions of crime and disorder. Contemporary research finds that a neighborhood’s Black racial composition is a better predictor of individuals perceiving a neighborhood as crime-ridden and disorderly than the observed presence of crime and disorder, particularly among White residents (Hinkle et al., 2023; Quillian & Pager, 2001; Sampson & Raudenbush, 2004). Racialized perceptions of neighborhood quality extend beyond crime and disorder to influence perceptions of school quality, housing value, and overall desirability, influencing not only how individuals interact with a neighborhood but also institutional gatekeepers and housing market intermediaries (Besbris & Korver-Glenn, 2023; Holme, 2002; Howell & Korver-Glenn, 2018, 2020; Korver-Glenn et al., 2021).

The use of race to determine neighborhood value and vitality is deeply entrenched in the U.S. housing industry, evident in industry rhetoric and practices throughout the twentieth century and most powerfully institutionalized in the practice of redlining (Babcock, 1932; Howell & Korver-Glenn, 2018, 2020; Markley, 2024b; Rothstein, 2017; Taylor, 2019; Winling & Michney, 2021). The Home Owners’ Loan Corporation (HOLC)’s creation of residential security maps is considered the impetus of redlining, a practice that denied Black neighborhoods opportunities for investment throughout the twentieth century (Rothstein, 2017). Though HOLC as a federal institution did not itself participate in the practice of redlining, it is responsible for creating the systematic neighborhood appraisal system which was later adopted by private lending institutions and other federal organizations such as the FHA and VA (Markley, 2024a; Michney, 2022; Michney & Winling, 2020). While growing evidence continues to expose how HOLC appraisers used a neighborhood’s racial composition to evaluate lending risk (Markley, 2024a, 2024b; Michney, 2022), we know relatively little about how crime influenced their risk assessments or how a neighborhood’s racial composition shaped how HOLC appraisers perceived and documented the presence of crime and disorder.

This question is particularly significant given contemporary research consistently shows that perceptions of neighborhood crime and disorder are deeply racialized (Hinkle et al., 2023; Quillian & Pager, 2001; Sampson & Raudenbush, 2004) and influential for neighborhood selection and property valuation (Krysan et al., 2009; Lewis et al., 2011). The conflation of race and criminality has deep historical roots in U.S. culture and law, with media rhetoric long depicting Black Americans as immoral, criminal threats and legal structures systematically criminalizing Black communities (Alexander, 2010). HOLC’s detailed neighborhood assessments offer a unique opportunity to investigate whether the spatial conflation of race and crime was embedded in early institutional rhetoric on neighborhood lending risk. The HOLC data provide a window into both how appraisers perceived and documented neighborhood crime and disorder, and how their perceptions, alongside actual observed crime rates, shaped their decisions on lending risk.

To investigate the historical relationship between race, crime, and lending risk, this paper combines several unique datasets on the city of Chicago, including HOLC’s residential security maps and the corresponding neighborhood area descriptions, 1940 Decennial Census data, Clifford Shaw et al.’s (1929) residence of male offenders map from 1920, and Frederic Thrasher’s (1927, 1936) Gangland map documenting the habitat of gangs between 1923 and 1926. Through systematic coding of the narratives within the neighborhood area descriptions, this study first identifies how often HOLC appraisers discussed the presence of neighborhood crime and disorder. Using traditional regression models, the paper then analyzes how observed neighborhood conditions, including local criminal activity and neighborhood racial composition, predicted the likelihood that HOLC appraisers mentioned the presence of crime and disorder in their neighborhood area descriptions. Finally, with traditional regression models, the paper investigates how both observed crime and perceptions of disorder, reflected through HOLC appraisers’ narratives, predicted their lending risk assessments. By investigating how both documented criminal activity and HOLC appraisers’ perceptions of crime and disorder influenced early mortgage risk assessments, this study provides new insights into the historical roots of neighborhood stigmatization and institutional disinvestment.

## Background

### Racialized Assessments of Neighborhood Value

Extensive contemporary and historical evidence documents the relevance of race for neighborhood desirability and property valuation (Babcock, 1932; Howell & Korver-Glenn, 2018, 2020; Krysan & Crowder, 2017; Krysan et al., 2009; Michney, 2022; Park & Burgess, 1925; Rothstein, 2017; Taylor, 2019). Both individual homeseekers as well as housing market intermediaries operate within an economic system of racial capitalism, which leverages neighborhood racial compositions to define economic value in the U.S. housing market (Dantzler et al., 2022; Markley, 2024b; Melamed, 2015; Robinson, 1983; Rucks-Ahidiana, 2021). In line with the existing racial hierarchy, housing in White neighborhoods is perceived as having a higher economic value than housing in comparable Black neighborhoods (Howell & Korver-Glenn, 2018, 2020; Korver-Glenn, 2018, 2021). The systematic devaluation of Black neighborhoods is rooted in early twentieth-century real estate ideology that viewed the presence of Black residents as a precursor to White flight, neighborhood racial turnover, and inevitable neighborhood decline (Babcock, 1924, 1932; Howell & Korver-Glenn, 2018, 2020; Park & Burgess, 1925).

HOLC’s neighborhood appraisal system helped to institutionalize early racist market preferences into formal risk assessment criteria, codifying the systematic devaluation of Black neighborhoods into standard lending practices. After the 1929 stock market crash, the federal government established HOLC in response to the mortgage crisis to refinance home mortgages and standardize home lending practices for the future (Michney, 2022; Michney & Winling, 2020). One way they standardized lending practices was by creating a neighborhood-based assessment of lending risk in the form of residential security maps, which were created for every city with a population of at least 40,000 residents. To create the maps, HOLC field agents worked intimately with local real estate and banking professionals who were more familiar with their city’s neighborhoods (Michney, 2022). The maps used a four-level classification system of lending risk defined as the neighborhood’s security grade, where A-grade neighborhoods were considered the ‘Best,’ B-grade neighborhoods were considered ‘Still Desirable,’ C-grade neighborhoods were considered ‘Definitely Declining,’ and D-grade neighborhoods were considered ‘Hazardous.’ The neighborhoods marked as ‘Hazardous’ and given a D-grade in lending risk were colored red in the maps—the namesake of redlining—where mortgage loans were ultimately discouraged (Jackson, 1985; Michney, 2022; Rothstein, 2017).

Although HOLC did not directly participate in the practice of redlining, as their lending had ceased before the creation of the maps, their neighborhood-based classification method helped formalize existing racial-spatial ideologies of value into an institutional framework for mortgage lending (Markley, 2024a). HOLC taught their neighborhood classification methods to private lending institutions throughout the country and shared their materials with the FHA (Michney, 2022; Winling & Michney, 2021). The Mapping Inequality project’s digitization of the HOLC materials (Nelson et al., 2016) has enabled scholars to document their profound institutional legacy. Research shows that cities that had the maps created for them saw increases in racial segregation (Faber, 2020b), and redlined neighborhoods experienced sustained disinvestment throughout the twentieth century (Aaronson, Hartley, & Mazumder, 2017; Appel & Nickerson, 2016; Faber, 2020a; Krimmel, 2017). These patterns persist today as residents living in historically redlined communities experience worse health outcomes (Krieger et al., 2020a, 2020b; Mujahid et al., 2021; Nardone et al., 2020a, 2020b; Nardone et al., 2020a, 2020b) and less opportunity for social mobility (Aaronson et al., 2021).

While scholars continue to document HOLC’s institutional legacy on the U.S.’s residential landscape, less attention has been paid to understanding how appraisers determined neighborhood lending risk classifications. Among existing research on HOLC’s classification methods, scholars have primarily focused on investigating the role of a neighborhood’s racial composition in determining lending risk, particularly decisions on redlining (Fishback et al., 2020; Hillier, 2005; Markley, 2024a; Michney, 2022; Michney & Winling, 2020; Rothstein, 2017; Winling & Michney, 2021). Although debates persist as to how centrally race influenced neighborhood classifications (see Markley, 2024a for the latest review), the HOLC materials still provide an unprecedented window into how racial-spatial ideologies shaped perceptions of real estate value during a period of active government oversight. The complex factors that shaped appraisers’ decision-making remain understudied, particularly how potentially racialized perceptions of neighborhood crime and disorder influenced decisions on lending risk.

### The Relevance of Neighborhood Crime and Disorder for Lending Risk

Perceptions of disorder and moral character have long shaped the social ranking of neighborhoods within cities, influencing where individuals believe a neighborhood falls in the status hierarchy. In one of the earliest examples of social cartography, Charles Booth’s (1889) poverty maps of London explicitly linked neighborhood social status to crime and disorder, describing the lowest classification of neighborhoods as “Lowest class. Vicious, semi-criminal.” The ascription of moral evaluations to his ecological classification system represents spatial differentiation not only by economic or racial status but also by perceived character and moral standing (Sampson, 2009). Judgments of disorder were effectively incorporated into a spatial hierarchy of neighborhood status. Similarly, HOLC’s neighborhood grading system helped create a hierarchy of neighborhood status based on housing valuation and lending risk. Given that the maps were meant to reflect underlying neighborhood desirability, it is feasible that assumptions about disorder, morality, and neighborhood character also shaped HOLC’s classification system of neighborhood lending risk.

Physical and social disorder serve as visible cues to neighborhood quality. Graffiti, abandoned cars, broken windows, and garbage littered throughout a neighborhood signal a neighborhood’s physical deterioration, while public intoxication, disorderly groups of young men, verbal harassment, and open solicitation for prostitution signal a neighborhood’s social deterioration (Sampson & Raudenbush, 1999). Visible signs of disorder, or “incivilities” (Hunter, 1985; Jacobs, 1961), suggest both a neighborhood’s inability or refusal to participate in city norms of presentation (Goffman, 1963; Sampson, 2012) and that there are less capable guardians on the street (Sampson & Groves, 1989). These conditions heighten perceived risk of victimization, generating a fear of crime that influences both insiders’ and outsiders’ willingness to interact in a neighborhood (Skogan, 1990).

Fear-induced withdrawal can trigger a self-reinforcing cycle of neighborhood decline. When residents perceive a neighborhood as disorderly, their confidence in their ability to respond to crime and disorder diminishes, leading to withdrawal from neighborhood life and even neighborhood outmigration (Peterson & Krivo, 2005; Rosen, 2017; Sampson, 2012; Skogan, 1990). Housing market instability emerges as existing residents move out and potential homebuyers are dissuaded from moving into neighborhoods they perceive as having higher crime rates (Doran & Burgess, 2012; Krysan & Crowder, 2017; Krysan et al., 2009; Lewis et al., 2011; Rosen, 2017). Local businesses also suffer as both residents and nonresidents avoid traversing the streets, leading to closures, relocations, and the opening of new businesses elsewhere (Wallace & Louton, 2018). Ultimately, how individuals perceive neighborhood disorder and crime is consequential for understanding neighborhood vitality (Sampson, 2012), perceiving disorder being a stronger predictor of future poverty rates than observed measures of disorder. Thus, disorder factors heavily into how both internal residents and outsiders evaluate and perceive neighborhood quality, determining their routine activities, travel patterns, and housing decisions (Krysan et al., 2009; Sampson, 2012; Skogan, 1990). HOLC appraisers likely also viewed disorder as a signal of neighborhood decline and a threat to current and future neighborhood valuation.

### Racialized Perceptions of Crime and Disorder

While perceptions of crime and disorder are informed by actual observed forms of crime and disorder, they are also influenced by the racial composition of a neighborhood (Sampson, 2012). In fact, the racial composition of a neighborhood tends to be a stronger predictor of whether individuals perceive disorder and crime than observed neighborhood conditions, with Blacker neighborhoods perceived as more crime-ridden and disorderly (Quillian & Pager, 2001; Sampson, 2012; Sampson & Raudenbush, 2004). Studies also show that White residents are generally more sensitive to perceiving a neighborhood as disorderly than Black residents (Hinkle et al., 2023) and are less likely to be satisfied with public safety when living in Black and Hispanic neighborhoods compared to when living in White neighborhoods (Cho & Ho, 2018). In the search for housing, White households are often unable to separate their stereotypical views on race from their perceptions of crime, disorder, and poverty when rating whether a neighborhood is a desirable place to live (Krysan & Crowder, 2017; Krysan et al., 2009; Lewis et al., 2011).

The endurance of such negative racialized perceptions of place stems from structural racism and sustained racial segregation that has systematically isolated many communities of color from resources and opportunities, while simultaneously reinforcing stereotypes that associate Black communities and individuals with crime, disorder, and moral decline (Alexander, 2010; Anderson, 2012, 2015; Bonilla-Silva, 2021; Krysan et al., 2009; Massey, 2004; Wacquant, 1993, 2008). These collective results point to the salience of race as an indicator of perceived neighborhood quality and desirability, including a neighborhood’s reputation for being disorderly and crime-ridden. While contemporary research clearly shows how race shapes perceptions of neighborhood disorder, we know less about how this relationship manifested historically. By analyzing HOLC’s neighborhood assessments of lending risk alongside historical data on criminal activity, this study examines whether race shaped appraisers’ perceptions of crime and disorder and how both their perceptions alongside actual crime influenced their lending risk decisions.

### The Case of Chicago

Chicago represents an ideal case study to investigate the historical relationship between race, crime, and neighborhood processes. The Chicago School of Sociology’s rich tradition of neighborhood research during the first half of the twentieth century produced unique data sources tracking observed patterns of crime and disorder. In particular, Shaw et al.’s (1929) residence of male offenders map and Thrasher’s (1927, 1936) survey of Chicago gangs provide detailed spatial documentation of the presence of crime and disorder in the years preceding the creation of the HOLC maps. Shaw et al.’s map of male offenders presents the place of residence for 7,541 alleged male offenders aged 17 to 75 placed in the Cook County jail in 1920 (Shaw et al., 1929). Thrasher’s *Gangland* map presents the location of 1,313 observed gangs in Chicago between 1923 and 1926 and is the product of his careful observation of gangs and their ‘natural habitat’ (Thrasher, 1936). While these maps of criminal activity predate the HOLC maps by over a decade (1920 and 1923–26 compared to 1939), they represent the best available measures of observed crime and disorder during this period.

Shaw and McKay (1942) demonstrated strong continuity in the spatial patterns of crime and disorder in Chicago during this period, suggesting these earlier maps remain valid proxies. Moreover, given the theoretical frameworks developed by Chicago School scholars, these maps capture both the social and spatial dimensions of neighborhood disorder. Shaw and McKay (1942)’s theory of social disorganization highlights how certain neighborhood conditions, including poverty, residential turnover, and ethnic heterogeneity, contribute to criminal activity by weakening social control. Thus, the spatial patterns documented in Shaw et al.’s male offenders’ place of residence and Thrasher’s gang territories reflect not just isolated incidents, but broader neighborhood social conditions that produce and sustain criminal activity. As such, these maps serve as theoretically grounded proxies for both the social and spatial dimensions of neighborhood disorder. While using data from an earlier period poses limitations, the detailed maps provide a unique opportunity to examine how observed patterns of crime and disorder influenced HOLC appraisers’ perceptions and assessments of lending risk.

## Methods

### Data

This study combines several data sources on the city of Chicago, including the historic HOLC maps and their corresponding neighborhood area descriptions; 1940 Decennial Census data; Clifford Shaw, Frederick Zorbaugh, Henry McKay, and Leonard S. Cottrell’s map of the place of residence of male offenders; and Frederic Thrasher’s gang activity map. The HOLC materials, stored in the National Archives, have been made publicly available and digitized by the Mapping Inequality group (Nelson et al., 2016).^1^ The maps and their corresponding neighborhood area descriptions are available as a downloadable geographic shapefile dataset. The data provide the lending risk assessment for each neighborhood, i.e., its security grade, along with the neighborhood area descriptions that HOLC appraisers filled out for each neighborhood they assessed. The neighborhood area descriptions were one-page memos that ensured the grading system was a standardized process both within and across cities. They contained multiple fill-in-the-blank prompts on the neighborhood conditions, such as the building age, availability of mortgage funds, and the class of occupants living in the neighborhood, as well as an additional paragraph-length space for HOLC appraisers to provide more explicit details on the neighborhood. The paragraph-length narratives allowed HOLC appraisers to discuss the local neighborhood conditions which they considered most relevant for the neighborhood’s security grade. These narratives reveal HOLC appraisers’ perceptions of the local neighborhood conditions and provide a glimpse into the conditions determining real estate valuation.

The 1940 Decennial Census data were collected by the U.S. Census Bureau to measure population and housing characteristics across the country. The Census Bureau presents this data in the form of census tracts which are often used to proxy neighborhoods. Shaw et al.’s map of male offenders provides data on the place of residence for 7,541 alleged male offenders aged 17–75 placed in the Cook County jail in 1920 (Shaw et al., 1929; Shaw, Behavior Research Fund, et al., 1929). The map was originally published in *Delinquency areas: a study of the geographic distribution of school truants, juvenile delinquents, and adult offenders in Chicago*. Thrasher’s Gangland Map provides data on the location of 1,313 observed gangs in Chicago between 1923 and 1926 (Thrasher, 1927, 1936). The map was originally published in *The gang: a study of 1,313 gangs in Chicago*. Thrasher and Shaw et al.’s original maps as well as the downloadable files are from the Social Scientists Map Chicago Collection in the University of Chicago Library’s Map Collection.^2^

The 1940 decennial census tract data are only available for neighborhoods which are an incorporated part of the city. As a result, the analysis is restricted to the Chicago HOLC neighborhoods with overlapping 1940 census data (*N* = 339 neighborhoods; 7 A-grade, 59 B-grade, 176 C-grade, and 97 D-grade).^3^ While this does exclude HOLC neighborhoods which were outside of the city limits, it still contains a large sample of neighborhoods which captures the criminal and racial processes which are the focus of this study given the historical concentration of racial groups and crime in the neighborhoods closer to the city center (Park & Burgess, 1925; Shaw & McKay, 1942). The final sample underrepresents A-grade neighborhoods and overrepresents D-grade neighborhoods.

To combine all four data sources, I used geospatial analytic techniques of spatial aggregation, with HOLC neighborhood boundaries serving as the primary spatial unit of analysis. For the census tract data, I used an area-weighted interpolation method. As the census tract boundaries did not perfectly align with the HOLC neighborhood boundaries, I calculated the proportion of each census tract that fell within each HOLC neighborhood. These proportions were then used as weights to aggregate demographic data. Thus, if 60% of a census tract’s area fell within a HOLC neighborhood, 60% of that tract’s demographic counts were attributed to that neighborhood. For tracts that overlapped with multiple HOLC neighborhoods, their demographic data were distributed proportionally based on the percentage of the areas overlapping. For the observed crime data, I used a point-in-polygon spatial join to aggregate Shaw et al.’s male offender locations and Thrasher’s gang locations within the HOLC neighborhood boundaries. This process involved dissolving point features from Shaw and Thrasher’s maps that fell within each HOLC polygon, allowing me to calculate the total count of both offenders and gangs in each HOLC neighborhood. Points falling exactly on neighborhood boundaries were assigned to a single neighborhood using GIS standard boundary rules. This spatial joining technique provides two quantitative measures of the historical concentration of criminal activity within each HOLC-defined neighborhood.

### Analytic Strategy

#### Identifying HOLC Appraisers’ Discussion of Crime and Disorder

To capture all discussions of crime and disorder, I iteratively and systematically examined all HOLC neighborhood area descriptions. The manual coding process involved reading each neighborhood area description in its entirety to understand the full context of crime-related discussions and flagging any neighborhood where crime or disorder were discussed. I coded a neighborhood as mentioning crime and disorder (= 1) when appraisers discussed issues related to vice, gangs, vandalism, disorder, crime, or similar concerns. This comprehensive approach allowed me to identify nuanced discussions of neighborhood disorder that might not have been captured through a text analysis method using key words alone. The manual coding for the neighborhoods included in the study sample revealed 22 total neighborhoods where crime and disorder were mentioned (19 in D-grade and 3 in C-grade neighborhoods),^4^ providing a robust measure of crime-related references in the HOLC documents.

#### Logistic and Multinomial Logistic Regression Analyses

After identifying how often HOLC appraisers discussed crime and disorder, I investigate how observed neighborhood conditions shaped HOLC narratives on the presence of crime and disorder using a logistic regression analysis. The analysis focuses on whether a neighborhood’s observed racial composition (according to census data) and observed criminal activity (according to the prior presence of gangs and male offenders in a neighborhood) predict the likelihood that HOLC appraisers mention crime and disorder in the neighborhood’s area description. I next investigate how both observed crime and perceptions of crime and disorder influenced decisions on lending risk using a multinomial logistic regression analysis.^5^ The analysis focuses on whether crime, measured both through observed data (according to the prior presence of gang and male offenders in a neighborhood) and perceived assessments (according to HOLC appraisers’ discussion of crime and disorder in the neighborhood area description), influenced HOLC appraisers’ decisions on lending risk, particularly redlining.

*Dependent Variables:* For the first analysis, the dependent variable is a dichotomous measure capturing whether HOLC appraisers mentioned the presence of crime and disorder in the neighborhood area description (= 1). These mentions are identified through an exhaustive manual coding of all neighborhood area descriptions discussed above. This measure also serves as an independent variable in the multinomial logistic regression analysis predicting lending risk. The dependent variable in the second analysis is the neighborhood’s security grade, i.e., its lending risk designation, which I operationalize as a categorical variable representing each security grade: A/B, C, and D. Given the limited sample of A-grade neighborhoods (*n* = 7), they are combined with B-grade neighborhoods.^6^

*Independent Variables:* The primary independent variables for both analyses are observed crime and neighborhood racial composition. Observed crime and disorder is proxied using the prior presence of male offenders and gang activity in a neighborhood. For both measures I create rates by aggregating the total number of offenders and gangs, respectively, and dividing them by the total population in the HOLC neighborhood before multiplying it by 1,000. The neighborhood’s observed racial composition is captured using data from the 1940 census, including both the percentage of Black residents and its squared term to account for Chicago’s highly segregated residential patterns. I also include the percentage of White foreign-born residents in a neighborhood as an additional demographic indicator.

*Controls:* I account for neighborhood socioeconomic conditions using 1940 census data, as research demonstrates that the socioeconomic and occupational class of neighborhood residents significantly influenced HOLC security grade assignments (Fishback et al., 2020).^7^ I include measures for the percentage of employed residents who are skilled workers (defined as residents employed as clerical/sales/kindred workers, craftsmen/foremen/kindred workers, or operatives) or laborers (defined as residents working as domestic service workers, other service workers, or laborers). I also account for the percentage of residents 14 years and older who are unemployed, and for the percentage of residents 25 years and older who have less than a high school education.

## Results

### Descriptive Statistics

Table 1 presents the descriptive statistics identifying how frequently HOLC appraisers discuss the presence of crime and disorder in the neighborhood area descriptions as well as the observed local neighborhood conditions from the census and criminal activity maps by security grade. In total, crime and disorder are mentioned in 22 neighborhoods, which is approximately 6.5 percent of all HOLC-graded neighborhoods. However, this discussion varies dramatically by security grade. Crime and disorder are mentioned in 20 percent of D-grade neighborhoods (*N* = 19), while they are never mentioned in A- or B-grade neighborhoods and are mentioned in less than 2 percent of C-grade neighborhoods (*N* = 3). These results align with expectations that crime and disorder are discussed more often in the lowest graded neighborhoods.

**Table 1:** Descriptive statistics by security grade in Chicago, *N* = 339

	D-Grade			C-Grade			B-Grade			A-Grade		
	Mean.	Min.	Max.	Mean.	Min.	Max.	Mean.	Min.	Max.	Mean.	Min.	Max.
Neighborhood crime and disorder												
Gang activity rate	1.22	0.00	66.67	0.11	0.00	3.92	0.01	0.00	0.23	0.00	0.00	0.00
Male offender rate	2.10	0.00	25.46	0.51	0.00	3.37	0.11	0.00	1.10	0.23	0.00	1.17
Perceived crime and disorder	0.20			0.02			0.00			0.00		
Local neighborhood conditions												
Percent black	9.56	0.00	96.26	0.83	0.00	90.71	0.66	0.00	20.02	0.81	0.11	1.26
Percent white foreign-born	20.60	0.52	50.00	18.67	1.86	40.87	15.11	6.92	33.33	11.81	8.21	19.60
Percent skilled workers	60.35	0.00	80.00	66.56	40.63	79.16	57.38	0.00	80.00	38.56	33.43	46.36
Percent laborers	24.12	0.00	55.91	14.75	0.00	45.45	12.02	0.00	34.39	18.40	9.16	29.77
Percent unemployed	14.14	0.00	40.44	8.55	0.00	25.00	5.89	0.00	25.00	3.58	2.77	4.79
Percent less than high school	69.43	24.27	100.00	53.54	21.04	83.33	40.44	18.67	66.67	27.33	22.55	34.07

Note: Crime rates are per 1,000 residents. The study sample includes 97 D-grade neighborhoods, 176 C-grade neighborhoods, 59 B-grade neighborhoods, and 7 A-grade neighborhoods. Gang activity rate data come from Thrasher's Gangland Map, which documents the location of 1,313 observed gangs in Chicago between 1923 and 26. Male offender rate data come from Shaw et al.’s map which documents the place of residence for 7,541 alleged male offenders aged 17 to 75 placed in the Cook County jail in 1920. Local neighborhood conditions data come from the 1940 U.S. decennial census. Perceived crime and disorder data come from the manual coding of HOLC neighborhood area description narratives identifying when HOLC appraisers mention the presence of crime and disorder

When crime and disorder are discussed, they are most often discussed in terms of the presence of vandalism (*N* = 16). For example, neighborhood D-38 states, “There is much vandalism throughout the area; the future appears hopeless,” and D-35 states, “Vandalism is prevalent and vacant units must maintain a caretaker.” Beyond vandalism, HOLC appraisers mention the presence of gangsters and dangerous elements (*N* = 5), brothels and shady institutions or gambling halls (*N* = 2), or a history and reputation for violence in the neighborhood (*N* = 3). While HOLC appraisers note the presence of vandalism or brothels as a sign of neighborhood disorder, they are also aware of the neighborhood’s sociohistorical contexts and its implications for neighborhood desirability.

The results for perceptions of crime and disorder align with the distribution of observed crime, as D-grade neighborhoods, on average, have the highest prevalence of previous gang activity and offenders residing in the neighborhood. On average, Black residents as well as laborers and residents who are unemployed or have less than a high school education are more prevalent in D-grade neighborhoods than C-, B-, and A-grade neighborhoods. White foreign-born residents and residents who are skilled workers are, on average, more prevalent in both D- and C-grade neighborhoods in comparison to B- and A-grade neighborhoods. The descriptive statistics are consistent with expectations that lower graded neighborhoods have higher crime rates, are more socioeconomically disadvantaged, and contain a larger presence of Black and foreign-born residents.

**Fig. 1:**
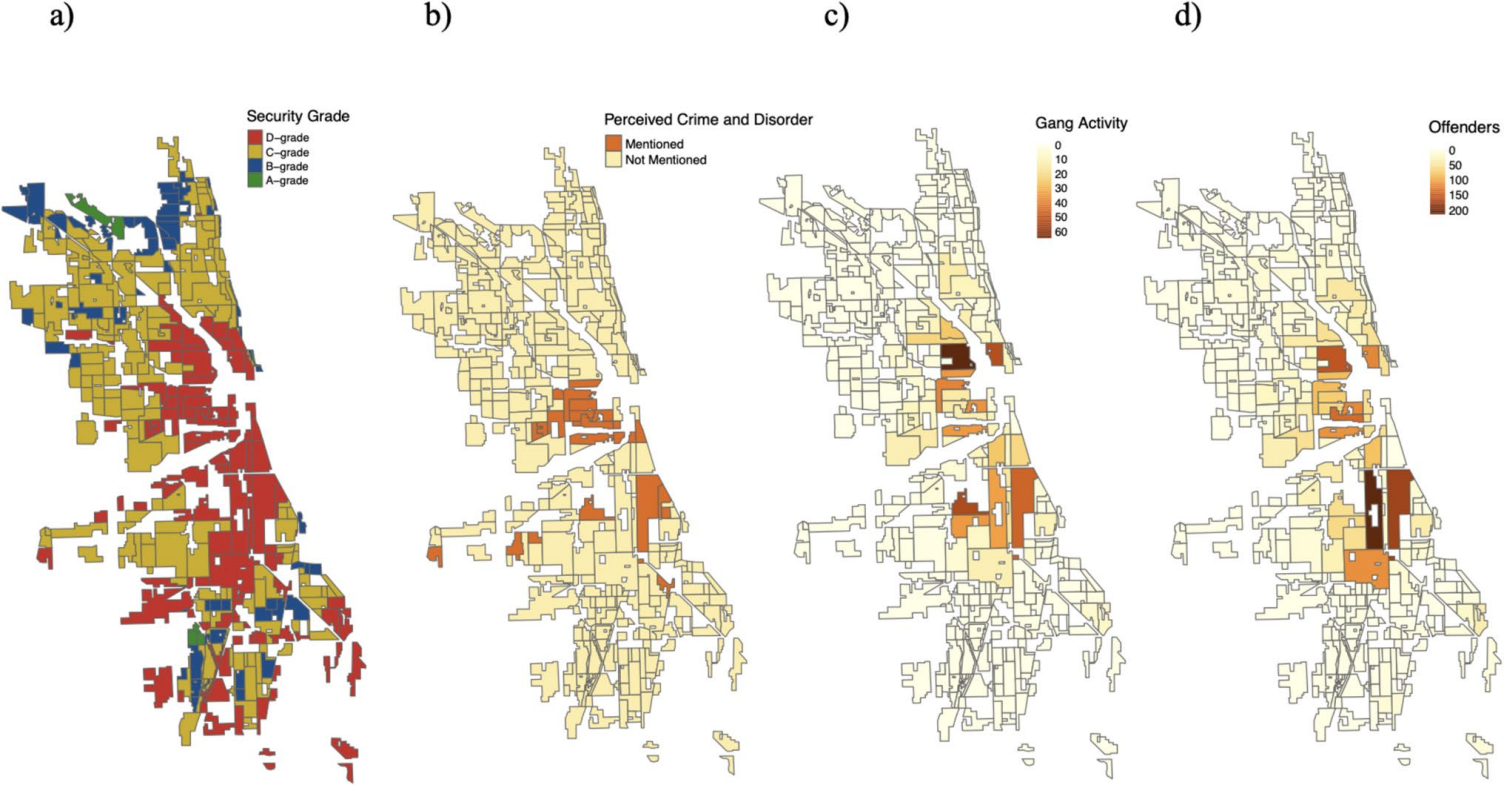
Spatial Distribution of HOLC **a** Security Grades, **b** Perceived Crime and Disorder, **c** Gang Activity, and **d** Male Offenders across Chicago Neighborhoods. The study sample includes 97 D-grade neighborhoods, 176 C-grade neighborhoods, 59 B-grade neighborhoods, and 7 A-grade neighborhoods. Gang activity data come from Thrasher’s Gangland Map, which documents the location of 1,313 observed gangs in Chicago between 1923 and 26. Male offender data come from Shaw et al.’s map which documents the place of residence for 7,541 alleged male offenders aged 17–75 placed in the Cook County jail in 1920. Perceived crime and disorder data come from the manual coding of HOLC neighborhood area description narratives identifying when HOLC appraisers mention the presence of crime and disorder

Figure 1 presents four maps presenting the spatial distribution of HOLC’s security grades representing lending risk assessments, perceived crime and disorder according to HOLC appraisers’ discussion of the presence of crime and disorder in the neighborhood area descriptions, prior gang activity documented by Thrasher between 1923 and 1926 (Thrasher, 1927, 1936), and the place of residence for male offenders aged 17–75 placed in the Cook County jail in 1920 (Shaw et al., 1929). The maps suggest that the neighborhoods with the highest level of lending risk tend to cluster in the city center and the neighborhoods immediately beyond it, coinciding with the areas that historically had a higher concentration of prior gang activity and the presence of male offenders in the 1920s. While there is a large degree of spatial overlap, HOLC appraisers’ written discussions of the presence of crime and disorder appear in fewer neighborhoods than the historical crime data would suggest. Nonetheless, there remains a strong correspondence between perceived crime, prior observed crime, and decisions on redlining.

#### Predicting Perceptions of Crime and Disorder

Table 2 presents the average marginal effects from the logistic regression analysis investigating how the observed neighborhood conditions, including local crime and racial composition, predict the likelihood HOLC appraisers discuss the presence of crime and disorder in a neighborhood area description.^8^ Model 1 includes only the observed crime rates, proxied as the prior presence of gangs and offenders in the neighborhood, while Model 2 incorporates the observed Black racial composition in addition to the controls for socioeconomic conditions. For interpretability, all percentage measures from the census data are operationalized where a 1-unit increase corresponds to a 10% increase, and the gang activity and offender residence rates are per 1,000 residents in the neighborhood.

**Table 2:** Average marginal effects of covariates from logistic regression predicting perceived crime and disorder in Chicago’s HOLC neighbourhood area descriptions, *N* = 339

	Model 1		Model 2	
	AME	SE	AME	SE
Neighborhood crime and disorder				
Gang activity rate	0.00	(0.00)	0.00	(0.00)
Male offender rate	0.02**	(0.01)	− 0.00	(0.00)
Local neighborhood conditions				
Percent black			0.04*	(0.02)
Percent white foreign-born			0.04	(0.02)
Percent skilled workers			− 0.01	(0.01)
Percent laborers			0.05*	(0.02)
Percent unemployed			0.00	(0.03)
Percent less than high school			0.01	(0.01)

A one-unit change for all local neighborhood conditions is equivalent to a 10% change. Gang activity and male offender rates are per 1,000. The study sample includes 97 D-grade neighborhoods, 176 C-grade neighborhoods, 59 B-grade neighborhoods, and 7 A-grade neighborhoods. Gang activity rate data come from Thrasher's Gangland Map, which documents the location of 1,313 observed gangs in Chicago between 1923 and 26. Male offender rate data come from Shaw et al.’s map which documents the place of residence for 7,541 alleged male offenders aged 17 to 75 placed in the Cook County jail in 1920. Local neighborhood conditions data come from the 1940 U.S. decennial census. Perceived crime and disorder data come from the manual coding of HOLC neighborhood area description narratives identifying when HOLC appraisers mention the presence of crime and disorder. Standard errors in parentheses; * p<0.05, ** p<0.01, *** p<0.001

Model 1 suggests that increasing the previous presence of male offenders in a neighborhood increases the likelihood that HOLC appraisers mention crime and disorder in the neighborhood area description. The prior presence of gangs, however, does not predict HOLC appraisers’ perceptions of crime and disorder. After accounting for the observed census characteristics, including the racial and socioeconomic composition, in Model 2, the relationship between observed crime and HOLC appraisers’ perceptions of crime disappears. Rather, increasing the Black racial composition and the percentage of workers employed as laborers increases the likelihood that HOLC appraisers mention crime and disorder. A 10 percent increase in a neighborhood’s Black racial composition is associated with a 4 percent increase in the predicted probability that HOLC appraisers mention crime and disorder. Similarly, a 10 percent increase in a neighborhood’s population employed as laborers is associated with a 5 percent increase in the predicted probability that HOLC appraisers mention crime and disorder. These findings suggest that the Black racial composition and occupational class of residents are more meaningful predictors of HOLC appraisers perceiving a neighborhood as disorderly or crime-ridden than observed measures of prior criminal activity.

#### Predicting Decisions on Lending Risk

Table 3 presents the average marginal effects from the multinomial logistic regression analysis investigating how observed neighborhood conditions, including local crime and racial composition, as well as HOLC appraisers’ perceptions of crime and disorder, predict HOLC appraisers’ assessments of lending risk.^9^ Model 1 includes only the observed measures of crime, proxied as the prior presence of gangs and offenders in the neighborhood. Model 2 incorporates the measure for perceived crime and disorder, indicating that HOLC appraisers mentioned the presence of crime and disorder in the neighborhood area description. Finally, Model 3 incorporates the observed Black racial composition in addition to the controls for socioeconomic conditions. Again, for interpretability, all percentage measures from the census data are operationalized, where a 1-unit increase corresponds to a 10% increase, and the gang activity and offender residence rates are per 1,000 residents in the neighborhood.

**Table 3:** Average marginal effects of covariates from multinomial logistic regression predicting lending risk of Chicago’s HOLC neighbourhoods, *N* = 339

	D-Grade						C-Grade						B-/A-Grade					
	Model 1		Model 2		Model 3		Model 1		Model 2		Model 3		Model 1		Model 2		Model 3	
	AME	SE	AME	SE	AME	SE	AME	SE	AME	SE	AME	SE	AME	SE	AME	SE	AME	SE
Neighborhood crime and disorder																		
Gang activity rate	0.30**	(0.11)	0.25*	(0.11)	0.08	(0.06)	0.53	(0.40)	0.53	(0.41)	0.44	(0.34)	− 0.83	(0.49)	− 0.78	(0.49)	− 0.52	(0.38)
Male offender rate	0.16***	(0.03)	0.15***	(0.03)	0.07**	(0.03)	0.10	(0.06)	0.11	(0.06)	0.07	(0.06)	− 0.26***	(0.07)	− 0.26***	(0.07)	− 0.13*	(0.06)
Perceived crime and disorder			0.41**	(0.14)	0.21	(0.13)			− 0.21	(0.14)	− 0.01	(0.13)			− 0.20***	(0.02)	− 0.20***	(0.02)
Local neighborhood conditions																		
Percent black					0.13**	(0.05)					− 0.08	(0.12)					− 0.05	(0.10)
Percent white foreign-born					– 0.09*	(0.04)					0.11	(0.06)					− 0.02	(0.05)
Percent skilled workers					– 0.15***	(0.03)					0.27***	(0.06)					− 0.12***	(0.03)
Percent laborers					– 0.07	(0.05)					0.17*	(0.08)					− 0.10	(0.06)
Percent unemployed					0.17*	(0.07)					− 0.10	(0.12)					− 0.07	(0.11)
Percent less than high school					0.13***	(0.02)					− 0.15***	(0.04)					0.02	(0.03)

A one-unit change for all local neighborhood conditions is equivalent to a 10% change. Gang activity and male offender rates are per 1,000. The study sample includes 97 D-grade neighborhoods, 176 C-grade neighborhoods, 59 B-grade neighborhoods, and 7 A-grade neighborhoods. Gang activity rate data come from Thrasher's Gangland Map, which documents the location of 1,313 observed gangs in Chicago between 1923 and 26. Male offender rate data come from Shaw et al.’s map which documents the place of residence for 7,541 alleged male offenders aged 17 to 75 placed in the Cook County jail in 1920. Local neighborhood conditions data come from the 1940 U.S. decennial census. Perceived crime and disorder data come from the manual coding of HOLC neighborhood area description narratives identifying when HOLC appraisers mention the presence of crime and disorder. Standard errors in parentheses; * p<0.05, ** p<0.01, *** p<0.001

Model 1 suggests that both measures of observed crime increase the likelihood a neighborhood is assigned a D-grade in lending risk. An increase of 1 gang or offender per 1,000 residents is associated with a 30% or 16%, respectively, increase in the predicted probability a neighborhood is assigned a D-grade in lending risk. An increase in the presence of male offenders also decreases the likelihood a neighborhood is assigned an A- or B-grade in lending risk. When HOLC appraisers’ perceptions of crime and disorder are included in Model 2, the observed measures of crime remain strong predictors of decisions on lending risk. HOLC appraisers’ perceptions of crime and disorder also serve as an additional predictor of lending risk. When appraisers mention the presence of crime or disorder in a neighborhood area description, it increases the predicted probability that they assign the neighborhood a D-grade in lending risk by 41% and decreases the predicted probability they assign it a B- or A-grade in lending risk by 20%.

When Model 3 includes the observed Black racial composition and socioeconomic conditions of the neighborhood, the relationship between crime and lending risk alters. Prior gang activity no longer influences the likelihood a neighborhood is assigned a D-grade in lending risk, nor do HOLC appraisers’ perceptions of crime and disorder in the neighborhood. Additionally, the magnitude of the relationship between the prior presence of offenders and lending risk is meaningfully reduced though remains a consistent predictor of lending risk. The full model finds a significant relationship between the observed Black racial composition and the likelihood a neighborhood is given a D-grade in lending risk. A 10 percent increase in the Black racial composition increases the predicted probability a neighborhood is redlined by 13 percent. A larger composition of residents who are unemployed and have less than a high school education also increases the likelihood a neighborhood is given a D-grade in lending risk, while neighborhoods with a higher concentration of White foreign-born residents have a decreased likelihood of D-grade assignment. Neighborhoods with a higher percentage of skilled workers, however, have a higher likelihood of being assigned a C-grade and a lower likelihood of being assigned an A/-B- and D-grade. A higher percentage of laborers is also associated with a higher likelihood of C-grade assignment, while a higher percentage of residents with less than a high school education is associated with a lower likelihood of C-grade assignment. These results indicate that the observed crime, race, and socioeconomic conditions of the neighborhood predict decisions on lending risk, while perceptions of crime and disorder do not.

#### Supplemental Analysis

I conduct supplemental analyses in the online Appendix to assess whether the results are sensitive to different measures of observed crime and disorder. Thrasher’s survey of gang activity and Shaw et al.’s offender data capture potentially distinct aspects of the criminogenic conditions representing neighborhood crime and disorder. The data on the place of residence of male offenders capture officially documented criminal activity through arrest records and court data. While the male offender rate may be biased due to geographic variation in enforcement and prosecution, it is a more direct measure of observable disorder through documented criminal justice system contact at the neighborhood level. The presence of gangs, in contrast, may not necessarily indicate visible criminal activity, but rather represent the neighborhood conditions that help produce gangs—disorder and social disorganization (Shaw & McKay, 1942; Thrasher, 1927). Thus, Appendix B presents sensitivity analyses on the inclusion of gang activity in the models. Tables B.1 and B.2 replicate the main tables when gang activity is excluded and find the conclusions are robust.

Tables B.3 and B.4 replicate the main tables using two alternative measures of gang activity. The alternative measures distinguish between gangs with and without clubrooms, following Thrasher’s (1927) original categorization. Thrasher noted that gangs with clubrooms were more formally organized, contained more resources, and sometimes even had political connections. The distinction thus reflects differences in resources as well as visibility and the types of activities in which gangs engaged. The results including gang activity by clubroom presence present the same substantive conclusions. However, it is notable that, when predicting lending risk, it is gangs without clubrooms that drive the relationship between prior gang activity and redlining, not gangs with clubrooms. While the findings should be interpreted with caution, the results may suggest that gangs with less resources are more likely to live in the worst-grade neighborhoods, but they may also suggest that gangs without clubrooms and less formal organization may have more visible street-level presence influencing assessments of neighborhood vitality.

## Discussion

Increasing evidence documents the profound influence of race on institutional practices of housing valuation and lending risk assessment throughout the twentieth century (Howell & Korver-Glenn, 2020; Markley, 2024a, 2024b; Michney, 2022; Rothstein, 2017; Taylor, 2019; Winling & Michney, 2021). Questions still remain, however, as to how racial-spatial ideologies shaped appraisers’ interpretations of local neighborhood conditions that would ultimately determine their risk assessments and establish lasting patterns of neighborhood valuation (Markley, 2024a). The HOLC archives offer a unique window into how racial prejudices filtered appraisers’ perceptions of neighborhood conditions, particularly crime and disorder. Contemporary research reveals that perceptions of neighborhood crime and disorder are deeply racialized and influential for neighborhood selection and property valuation (Krysan & Crowder, 2017; Krysan et al., 2009; Quillian & Pager, 2001; Sampson, 2012; Sampson & Raudenbush, 2004). Examining early institutional practices can thus offer insights as to how the conflation of race and crime, deeply rooted in the U.S.’s cultural and legal history (Alexander, 2010), became spatially embedded into how individuals understand and navigate the city. Using the case of Chicago, this study investigates how appraisers perceived and documented neighborhood crime and disorder, and how these perceptions alongside actual observed local conditions of crime and disorder shaped their risk assessments. The analysis reveals the historical antecedents of racialized perceptions of crime that would become deeply embedded in the U.S. residential landscape.

Analysis of the HOLC neighborhood area description narratives revealed that HOLC appraisers discussed the presence of crime and disorder in 6.5 percent of all HOLC-graded neighborhoods in the sample.^10^ HOLC appraisers noted the presence of crime and disorder overwhelmingly in D-grade neighborhoods (20 percent of all D-grade area descriptions). In contrast, the presence of crime and disorder was never mentioned when describing any A- or B-grade neighborhoods and appeared in only 2 percent of C-grade neighborhoods. When HOLC appraisers mentioned crime and disorder, they most often noted the presence of vandalism, although they also noted the presence of dangerous elements and gangers, brothels and gambling institutions, as well as when the neighborhood had a history for vice. These findings align with insights on the role of both physical and social disorder for informing how individuals perceive neighborhood quality (Hunters, 1985; Jacobs, 1961; Sampson, 2012; Sampson & Raudenbush, 1999; Skogan, 1990), suggesting that neighborhood incivilities were considered relevant to neighborhood valuation as housing market intermediaries documented their presence while making decisions on neighborhood lending risk. The results also demonstrate a persistent pattern of perceived crime and disorder being concentrated in the worst graded neighborhoods (Booth, 1889; Sampson, 2009).

There was strong correspondence between observed crime and perceived crime when comparing the map visualizations and examining the bivariate associations. However, once the observed racial and socioeconomic conditions of the neighborhood were accounted for, the Black racial and laborer occupational composition of the neighborhood predicted HOLC appraisers perceiving crime and disorder better than prior gang activity and the male offender rate. The findings are consistent with contemporary literature suggesting that the Black racial composition of a neighborhood is a better predictor of neighborhoods being perceived as crime-ridden and disorderly than observed conditions of crime and disorder (Quillian & Pager, 2001; Sampson & Raudenbush, 2004). The findings illustrate how ascriptions of morality and criminality have long served to mark and marginalize Black communities, serving to maintain racial boundaries and hierarchies (Anderson, 2012; Bonilla-Silva, 1997, 2021; Wacquant, 1993).

Both observed and perceived measures of crime and disorder predicted HOLC appraisers’ decisions on redlining before accounting for the local neighborhood conditions. However, once the observed racial and socioeconomic conditions of the neighborhood were accounted for, perceived crime and disorder no longer mattered for lending risk. Rather, the Black racial composition, prior male offender rate, and various socioeconomic indicators predicted redlining decisions. These results suggest that while HOLC appraisers’ perceptions of crime and disorder were racially motivated, their biased perceptions did not exert a unique, independent influence on their decisions to redline Black neighborhoods. Instead, racial bias was already fundamentally embedded in the real estate ideology determining neighborhood valuation and lending risk during the twentieth century (Markley, 2024a, 2024b; Michney, 2022; Taylor, 2019). These findings align with contemporary research on residential decision-making, which identifies socioeconomic conditions, crime and disorder, and racial composition as influential for neighborhood desirability (Krysan et al., 2009; Lewis et al., 2011). However, the historical insignificance of perceived crime and disorder contrasts with modern studies demonstrating the importance of perceptions for neighborhood vitality (Hwang & Sampson, 2014; Sampson, 2009, 2012).

This study contains several limitations to advance upon in future research. First, this study focuses exclusively on Chicago. While Chicago’s unique historical data on crime enable this analysis, patterns of crime and racial segregation vary across the U.S. cities. Future research would benefit from exploring regional variation in the relationship between crime, race, and neighborhood valuation. Second, the measures used to represent the observed conditions of crime and disorder should be interpreted with caution. Both measures are quite dated, representing prior criminal activity in 1920 and 1923–1926, while the HOLC maps were created in 1939. Additionally, neither measure is a perfect representation of criminal activity. The male offender rate may be biased to represent geographic variation in enforcement and prosecution and represents their residential addresses, not the location where the crime took place. The presence of gangs may also not necessarily indicate visible or active criminal activity. Nonetheless, both measures can be interpreted as representing the neighborhood conditions that help produce offenders and gangs—disorder and social disorganization (Shaw & McKay, 1942; Thrasher, 1927). Additionally, Shaw and McKay (1942) demonstrate a strong continuity in the historic presence of crime in the city of Chicago during this same period, suggesting that the dated maps remain valid proxies. Thus, while the measures for observed crime and disorder are limited, they are the best currently available data to investigate the relationship between race, crime, and redlining.

Third, the analysis does not account for spatial relationships in the data. Both crime and demographic conditions tend to cluster together in space, suggesting potential spatial autocorrelation and spatial spillover effects across neighborhood borders. Additionally, the spatial aggregation of census tract boundaries into HOLC neighborhood boundaries, which do not perfectly overlap, may introduce measurement error through the modifiable areal unit problem (MAUP). Future research should explore the spatial dynamics of redlining decisions and the sensitivity of the results to different spatial aggregations. Fourth, the analytic approach cannot support strict causal claims about the relationship between race, crime, and redlining. There are likely unmeasured factors that influence HOLC appraisers’ decisions on lending risk beyond race and crime, especially infrastructure quality, business activity, and the presence of neighborhood amenities. Future research should explore the features of the built environment shaping perceptions on neighborhood desirability, lending risk, and market value.

Overall, this study reveals a complex historical relationship between race, crime, and institutional decision-making on lending risk and neighborhood valuation. Evidence from the HOLC materials indicates that housing appraisers perceived crime and disorder using a racial lens, more often discussing the presence of crime and disorder in Black neighborhoods. In contrast with contemporary research, however, HOLC appraisers’ biased perceptions of crime and disorder did not exert an independent influence on their decisions regarding lending risk. These results reflect the historical reality of structural racism and racial capitalism, where White homeowners and real estate and banking professionals considered the presence of Black neighbors as detrimental for home values and were given explicit directives to consider race in housing valuation (Babcock, 1924, 1932; Markley, 2024a, 2024b; Michney, 2022; Rothstein, 2017; Taylor, 2019). Modern housing discrimination, however, often operates through coded language about neighborhood conditions, where discussions of crime and disorder serve as proxies for discussions of a neighborhood’s racial composition (Krysan & Crowder, 2017; Krysan et al., 2009). The results enhance our understanding of how racial bias in housing markets has evolved from explicit discrimination to more subtle forms of exclusion. Additionally, to my knowledge, this is the first study to investigate the relationship between crime and lending risk, finding that prior criminal activity in a neighborhood influenced decisions on redlining. These results have important implications for understanding neighborhood valuation, as neighborhood conditions not captured in administrative census data were also relevant for neighborhood appraisals. This study offers unique insights into how institutional gatekeepers considered the duality of crime and race when making decisions on lending risk and neighborhood valuation.

## Conflict of interest

The author declares that they have no conflict of interest.

## Supplementary Information

Below is the link to the electronic supplementary material.

Supplementary file1 (DOCX 707 KB)

## Footnotes

1 The HOLC data can be accessed here https://dsl.richmond.edu/panorama/redlining/

2 The maps can be accessed here https://www.lib.uchicago.edu/e/collections/maps/chisoc/

3 This compares to the full HOLC sample of 580 Chicago neighborhoods.

4 For the full sample of 580 neighborhoods, crime and disorder were discussed in 32 area descriptions, 5.5% of all area descriptions. The distribution of perceived crime and disorder across security grades was the same, never occurring in A- and B-grade neighborhoods, and occurring in 20% of D- and 2% of C-grade neighborhood area descriptions.

5 Though the neighborhood security grade is an ordinal variable (A/B, C, D), diagnostic tests revealed violations of the proportional odds assumption, indicating that an ordinal logistic regression was inappropriate for the data.

6 The results are consistent whether A-grade neighborhoods are dropped or combined with B-grade neighborhoods.

7 To assess potential multicollinearity between neighborhood racial composition, crime, and socioeconomic status indicators, I calculate Variance Inflation Factors (VIF) for the full models. The VIF values indicate no concerning levels of collinearity among these predictors.

8 The log odd results are presented in the Appendix.

9 The log odd results are presented in the Appendix.

10 In the full sample of 580 Chicago neighborhoods, HOLC appraisers discuss crime and disorder in 5.5 percent of the neighborhood area descriptions.
