# Supplementary material for: Race, Crime, and Lending Risk in Chicago: The Relevance of Crime and Disorder for HOLC’s Neighborhood Assessments
Source: Race Soc Probl. 2025 Aug 27;17(4):307–21. doi: 10.1007/s12552-025-09442-4 (PMC12605564; doi:10.1007/s12552-025-09442-4)
Supplement: Supplementary file 1 — Supplementary file1 (DOCX 707 KB) [file 12552_2025_9442_MOESM1_ESM.docx]

**List of Appendices**

Appendix A. The Log Odds Results for Main Tables

Appendix B. Sensitivity Analysis on the Inclusion of Gang Activity

**Appendix A. The Log Odds Results for Main Tables**

Appendix A presents the log odds that correspond with the results presented in Tables 2 and 3. D-grade neighborhoods serve as the reference category. As the results are discussed in the paper, they are not elaborated on here.

**Appendix B. Sensitivity Analysis on the Inclusion of Gang Activity**

Appendix B presents sensitivity analyses on the inclusion of gang activity in the models. Tables B.1 and B.2 replicate the main tables when gang activity is excluded, finding the main conclusions are robust. The male offender rate continues to predict HOLC appraisers’ perceptions of crime and disorder before controlling for the local neighborhood conditions. Once these conditions are included, only the Black racial composition and presence of laborers in the neighborhood increase the likelihood that appraisers mention the presence of crime and disorder. Similarly, the results are robust predicting HOLC appraisers’ lending risk assessments. A higher male offender rate increases the likelihood HOLC appraisers redline the neighborhood, as does a larger Black racial composition, while perceptions of crime and disorder do not.

Tables B.3 and B.4 replicate the main tables using two alternative measures of gang activity. The alternative measures distinguish between gangs with and without clubrooms, following Thrasher’s (1927) original categorization. Thrasher noted that gangs with clubrooms were more formally organized, contained more resources, and sometimes even had political connections. The distinction thus reflects differences in resources as well as visibility and the types of activities in which gangs engaged. The alternative measures are both continuous measures representing rates per 1,000. The results including gang activity by clubroom presence present the same substantive conclusions. However, it is notable that, when predicting lending risk, that it is gangs without clubrooms that drive the relationship between prior gang activity and redlining, not gangs with clubrooms. While the findings should interpretated with caution, the results may suggest that gangs with less resources are more likely to live in the worst grade neighborhoods, but they may also suggest that gangs without clubrooms and less formal organization may have more visible street-level presence influencing assessments of neighborhood vitality.
